# Patients’ Preferences for Surgical Management of Esophageal Cancer: A Discrete Choice Experiment

**DOI:** 10.1007/s00268-015-3148-8

**Published:** 2015-07-14

**Authors:** Esther W. de Bekker-Grob, Eva J. Niers, J. Jan B. van Lanschot, Ewout W. Steyerberg, Bas P. L. Wijnhoven

**Affiliations:** Department of Public Health, Erasmus MC - University Medical Centre Rotterdam, PO Box 2040, 3000 CA Rotterdam, The Netherlands; Department of Surgery, Erasmus MC - University Medical Centre Rotterdam, Rotterdam, The Netherlands

## Abstract

**Background:**

Obtaining insight into patients’ preferences is important to optimize cancer care. We investigated patients’ preferences for surgical management of esophageal cancer.

**Methods:**

We conducted a discrete choice experiment among adult patients who had undergone esophagectomy for adenocarcinoma or squamous cell cancer of the esophagus. Patients’ preferences were quantified with regression analysis using scenarios based on five aspects: risk of in-hospital mortality, risk of persistent symptoms, chance of 5-year survival, risk of surgical and non-surgical complications, and hospital volume of esophageal cancer surgery.

**Results:**

The response rate was 68 % (104/142). All aspects proved to influence patients’ preferences (*p* < 0.05). Persisting gastrointestinal symptoms and 5-year survival were the most important attributes, but preferences varied between patients. On average, patients were willing to trade-off 9.5 % (CI 2.4–16.6 %) 5-year survival chance to obtain a surgical treatment with 30 % lower risk of gastrointestinal symptoms, or 8.1 % (CI 4.0–12.2 %) 5-year survival chance for being treated in a high instead of a low-volume hospital.

**Conclusions:**

Patients are willing to trade-off some 5-year survival chance to achieve an improvement in early outcomes. Given the preference heterogeneity among participants, the present study underlines the importance of a patient-tailored approach when discussing prognosis and treatment.

## Introduction

Esophageal cancer is an aggressive disease, with a 15 % overall 5-year survival rate [1]. Surgery (i.e., esophagectomy) combined with neoadjuvant therapy offers the best chances for cure but is associated with significant mortality and morbidity rates [2]. Esophagectomy can cause troublesome and persistent gastrointestinal problems and is associated with diminished health-related quality of life [3, 4].

From the patients’ perspective, optimal surgical management of esophageal cancer weights aspects such as survival chance and (non-)surgical complications, and the experience of the hospital to conduct esophagectomies. To optimize cancer care, it is important for health care providers and policy makers to obtain insight into patients’ preferences towards surgical management for esophageal cancer, and to elicit the trade-offs that patients make. This insight is not only relevant to meet patients’ expectations, but also to provide high quality and responsive care [5]. However, quantitative studies investigating patients’ preferences for surgical management of esophageal cancer are lacking.

This study investigated patients’ preferences for surgical management of esophageal cancer and elicited the trade-offs that patients make. We hereto performed a discrete choice experiment (DCE), a quantitative approach that is increasingly used in healthcare [6].

## Materials and methods

### Study sample and elicitation mode

We identified patients who had undergone an esophagectomy for adenocarcinoma or squamous cell cancer of the esophagus at the Erasmus MC, University Medical Centre Rotterdam, The Netherlands. Patients who were alive and did not have difficulties in understanding the Dutch language were invited to participate in the study. The questionnaire and a prepaid return envelope were mailed to the patients. After 3 weeks, non-responders were sent a reminder. Written informed consent was obtained from all patients. The study was approved by the Medical Ethical Committee of the Erasmus MC—University Medical Centre Rotterdam (MEC-2011-217).

### Discrete choice experiment (DCE)

In DCEs, it is assumed that a medical intervention, such as a surgical treatment, can be described by its characteristics (attributes; e.g., risk of in-hospital mortality), and that patient’s preferences for an intervention are determined by the levels of the attributes (e.g., for risk of in-hospital mortality: 2, 5, and 10 %) [7]. The relative importance of attributes and the trade-offs that patients make between them can be assessed when patients are offered a series of choices between treatment alternatives that have different combinations of attribute levels (see “Appendix” for an example of a choice set) [8].

### Attributes and attribute levels

The choice of the most relevant attributes of surgical management of esophageal cancer and their attribute levels was based on literature [9–12], interviews with experienced upper gastrointestinal surgeons (*n* = 3), and patients who had undergone esophagectomy for esophageal cancer (*n* = 6; i.e., the target group). These were in-hospital mortality (chance of dying in the hospital after esophagectomy), persistent gastrointestinal (GI) symptoms (development of symptoms postoperatively including dysphagia, feeling of early fullness, nausea, regurgitation, and diarrhea), 5-year survival (chance to be alive without recurrence 5 years after esophagectomy), morbidity (surgical and non-surgical complications during hospital stay requiring medical or surgical treatment), and hospital volume (annual number of esophagectomies per hospital) (Table 1).

**Table 1:** Attributes and attribute levels for surgical management of esophageal cancer

Attributes	Levels
In-hospital mortality	2 %
	5 %
	10 %
Persistent gastrointestinal symptoms	10 %
	40 %
	80 %
5-year survival after esophagectomy	20 %
	35 %
	50 %
Risk for postoperative complications (morbidity)	20 %
	40 %
	60 %
Hospital volume	Low (<10 esophagectomies per year)
	Medium (10–40 esophagectomies per year)
	High (>40 esophagectomies per year)

### Study design and questionnaire

The combination of five attributes with three levels each resulted in 243 (3^5^) possible alternatives for surgical management of esophageal cancer. It is not feasible to present a single individual with all these alternatives. We therefore reduced the design in such a way that at least all main effects could be estimated. NGene software (http://www.choice-metrics.com/) was used, which is capable of generating designs that are highly efficient (i.e., maximizing D-efficiency or minimizing D-error). As a result, 24 choice sets divided over two versions of the questionnaire were constructed [13]. Each choice set included two surgical alternatives (“Appendix”). Patients were asked to consider the two alternatives in each choice set as realistic alternatives and to choose the alternative that appealed most to them.

Each questionnaire started with a detailed description of the attributes and their levels. The main part of each questionnaire comprised 12 choice sets. Furthermore, the following data were collected: age at completing the questionnaire, gender, level of education, and household situation. The questionnaire was pilot tested in an interview-based setting (*n* = 9) to check for any problems in interpretation and face validity.

### Statistical analyses

The DCE was analyzed by taking each choice among the two surgical alternatives as an observation. The observations were analyzed by a panel mixed logit model to obtain insight into patients’ preferences and to take preference heterogeneity as well as correlation between the choice tasks completed by each individual into account (since each respondent completed 12 choice tasks) [8]. After testing for linear effects of each continuous attribute, the following utility model was estimated:

1 $$V = \beta_{0} + \beta_{1} {\text{MORTALITY}} + \beta_{2} {\text{LONGTERM}}\_{\text{COMPLICATIONS}} + \beta_{3} {\text{FIVE}}\_{\text{YEAR}}\_{\text{SURVIVAL}} + \beta_{4} {\text{MORBIDITY + }}\beta_{5} {\text{HOSPITAL}}\_{\text{VOLUME}}\_{\text{MEDIUM}} + \beta_{6} {\text{HOSPITAL}}\_{\text{VOLUME}}\_{\text{HIGH}}$$

in which the coefficients for all attributes were treated as normally distributed random parameters.

*V* represents the utility (preference score) derived for an esophageal surgical alternative. *β*_0_ is a constant and *β*_1_–*β*_6_ are coefficients that indicate the relative weight individuals place on a certain attribute (level). The sign of a coefficient reflects whether the attribute has a positive or negative effect on the utility. The value of each coefficient represents the importance respondents assign to an attribute or attribute level. A statistically significant coefficient (*p* ≤ 0.05) indicates that individuals differentiated between one attribute (or attribute level) and another in making their choices in the DCE. A priori, we expected all attributes to be important. We expected that only the attribute ‘5-year survival after esophagectomy’ and the attribute levels of ‘hospital volume’ would have a positive effect (i.e., a positive sign).

We generated relative utility (preference) scores of esophagectomy scenarios based on the estimated coefficients. The higher a relative utility score, the stronger the preference for that particular scenario. Absolute values of *V*, however, have no direct interpretation [14]. We calculated the preference score (i.e., the mean utility) for a base case scenario, representing esophagectomy in a medium volume hospital with a 5 % risk of in-hospital mortality, 40 % risk of persistent GI symptoms, 35 % chance of 5-years survival, and 40 % risk of complications (morbidity). By changing one or more attribute levels, we obtain insight how each attribute systematically affects the utility score (and rank) relative to the base case. We took all preference heterogeneity into account in calculating the mean utility [15]. Finally, to investigate the willingness to trade-off 5-year survival to achieve an improvement in one of the other attributes, we calculated the ratios between the coefficients of the attributes with the attribute ‘5–year survival’ as the denominator.

## Results

### Respondents

The response rate to the questionnaire was 104/142 (68 %) and 97/104 (93 %) completed the DCE task. These respondents had a mean age of 64 years (SD = 8.8), 70 % were men, 28 % had a higher educational level, and 81 % lived together with a partner or family member (Table 2).

**Table 2:** Characteristics of respondents, who completed the discrete choice experiment

Characteristics	Respondents (*n* = 97)	
	Mean	SD
Age (years)	64	8.8

Characteristics	*n*	%
Age group (years)		
<60	26	27
60–69	46	47
≥70	25	26
Male gender	68	70
Educational level		
Lower education	31	32
Intermediate education	30	31
Higher education	27	28
Missing	9	9
Household		
With partner/family member	79	81
Single	13	13
Missing	5	5

### DCE results

All five attributes proved to influence patients’ preferences for surgical management of esophageal cancer (*p* < 0.05; Table 3). The positive or negative directions of the coefficients were consistent with our a priori hypotheses. The positive sign given to the coefficient ‘5-year survival’ indicated that patients preferred a surgical management of esophageal cancer generating an increase of 5-year survival after esophagectomy over surgical management that generates a lower chance of 5-year survival. The negative signs for in-hospital mortality, persistent GI symptoms, and morbidity indicated that patients preferred a surgical management of esophageal cancer with a low risk of negative side effects. Patients significantly preferred a high-volume hospital over a low-volume hospital, even after statistically adjusting for the other attributes related to medical care (in-hospital mortality, persistent GI symptoms and morbidity). All estimated standard deviations were significant, which indicated preference heterogeneity among patients for several attributes of surgical management of esophageal cancer.

**Table 3:** Patients’ preferences for surgical management of esophageal cancer based on a panel mixed logit model

Attributes	Coefficient	Mixed logit value	95 % CI
Constant	Mean	0.10	(−0.20 to 0.40)
	SD	0.74*	(0.29 to 1.20)
In-hospital mortality (per 10 %)	Mean	−3.67*	(−5.03 to −2.30)
	SD	1.46*	(0.57 to 2.35)
Persistent GI symptoms (per 10 %)	Mean	−0.74*	(−0.95 to −0.53)
	SD	0.38*	(0.24 to 0.51)
5-year survival (per 10 %)	Mean	2.33*	(1.12 to 3.55)
	SD	2.14*	(1.36 to 2.92)
Morbidity (per 10 %)	Mean	−0.67*	(−0.90 to −0.45)
	SD	0.38*	(0.21 to 0.54)
Hospital volume			
Low (omitted)	Mean	−1.00*	(−1.40 to −0.59)
	SD		
Medium	Mean	0.11	(−0.16 to 0.38)
	SD	0.67*	(0.38 to 0.96)
High	Mean	0.89*	(0.55 to 1.22)
	SD	0.92*	(0.52 to 1.31)

Effects coded variable used for hospital volume. Normal distribution for random coefficients used on all attributes. The value of the omitted term equals the negative sum of the coefficients of the included attribute levels

*GI* gastrointestinal

* *p* < 0.05 for statistical significance

### Utility scores and patients’ ranks for various surgical management scenarios

The preference score (i.e., the mean utility) for the base case scenario (esophagectomy in a medium volume hospital with 5 % in-hospital mortality, 40 % persistent GI symptoms, 35 % 5-year survival, and 40 % morbidity) was 0.67 (Table 4). In other words, the base case scenario was ranked 13 on a list of 21 (hypothetical) surgical treatments for esophageal cancer (Table 4). Especially an increased chance of 5-year survival from 35 to 50 %, or a decrease in risk of long-term GI symptoms from 40 to 10 % had a relatively large positive impact on the utility score and thus on the ranking score (rank 3 and 5, respectively, compared to rank 13 for the base case, all else being equal) (Table 4, scenarios 3 and 2, respectively). A high hospital volume instead of a medium volume hospital had a relatively small positive impact on the ranking (rank 12 compared to rank 13 for the base case, all else being equal) (Table 4, scenario 5). However, surgical managements that patients preferred were not automatically associated with optimal levels for chance of 5-year survival and (absence of) persistent GI symptoms. Holding the chance of 5-year survival constant, patients ranked a surgical management with a higher risk of long-term GI symptoms higher than a surgical management with a lower risk of long-term GI symptoms as long as the levels for risk of in-hospital mortality, risk of surgical complications as well as hospital volume were more optimal (Table 4; scenario 15 compared with scenarios 3 or 14). Similarly, holding the risk of persistent GI symptoms constant, patients ranked a surgical management with a lower chance of 5-year survival higher than a surgical management with a higher chance of 5-year survival as long as the levels for risk of in-hospital mortality, risk of surgical complications as well as hospital volume were more optimal (Table 4; scenario 15 compared with scenarios 2 or 13). This means that patients accepted a less effective surgical treatment or a higher risk of persistent GI symptoms, if esophagectomy took place in a higher volume hospital with a lower risk of in-hospital mortality and morbidity.

**Table 4:** Patients’ ranks and utility scores of hypothetical surgical treatments for esophageal cancer scenarios

	In-hospital mortality (%)	Persistent GI symptoms (%)	5-year survival (%)	Morbidity (%)	Hospital volume	Utility score	Difference in utility score compared to base case	Rank
Base case	5	40	35	40	Medium	0.67	0.00	**13**
One level improvement								
Scenario 1	**2**	40	35	40	Medium	1.90	1.23	10
Scenario 2	5	**10**	35	40	Medium	3.00	2.33	5
Scenario 3	5	40	**50**	40	Medium	4.34	3.67	3
Scenario 4	5	40	35	**20**	Medium	2.13	1.46	8
Scenario 5	5	40	35	40	**High**	1.47	0.80	12
One level worsening								
Scenario 6	**10**	40	35	40	Medium	−1.07	−1.74	17
Scenario 7	5	**80**	35	40	Medium	−2.08	−2.75	18
Scenario 8	5	40	**20**	40	Medium	−2.72	−3.39	20
Scenario 9	5	40	35	**60**	Medium	−0.49	−1.16	15
Scenario 10	5	40	35	40	**Low**	−0.31	−0.98	14
Mixed level change								
Scenario 11	**2**	**10**	**50**	**20**	**High**	9.73	9.06	1
Scenario 12	**10**	**10**	**50**	**20**	**High**	6.71	6.04	2
Scenario 13	**2**	**10**	35	40	Medium	4.07	3.40	4
Scenario 14	5	**80**	**50**	**20**	Medium	2.89	2.22	6
Scenario 15	**10**	**10**	**50**	**60**	**Low**	2.21	1.54	7
Scenario 16	**2**	40	35	**20**	**Low**	2.08	1.41	9
Scenario 17	5	40	**50**	**60**	**High**	1.49	0.82	11
Scenario 18	5	40	**20**	**20**	**High**	−0.62	−1.29	16
Scenario 19	**10**	**10**	**20**	40	Medium	−2.37	−3.04	19
Scenario 20	**10**	**80**	**20**	**60**	**Low**	−9.99	−10.66	21

Bold values highlight the change in attribute levels compared to the base case scenario

### Willingness to trade-off 5-year survival

Based on the expressed preferences, patients showed their willingness to trade-off their chance of 5-year survival to achieve an improvement in one level of the other attributes of surgical management of esophageal cancer (Table 5). On average, patients were willing to trade-off 3.9 % (CI 0.6–7.3 %) 5-year survival chance to obtain a surgical treatment with a 2.5 % lower absolute risk of in-hospital mortality. Patients were willing to trade-off 9.5 % (CI 2.4–16.6 %) or 5.8 % (CI 1.2–10.4 %) 5-year survival to obtain a surgical treatment with 30 % lower absolute risk of GI symptoms or 20 % lower absolute risk of morbidity, respectively. For receiving a surgical treatment in a high-volume hospital instead of a low-volume hospital, patients were willing to trade-off 8.1 % (CI 4.0–12.2 %) 5-year chance, keeping other attributes constant.

**Table 5:** Willingness to trade-off 5-year survival chance to achieve an improvement in one of the surgical management of esophageal cancer attributes based on panel mixed logit model

	Average (*n* = 97)		Interpretation note
	Willingness to trade 5-year survival (%; CI)		To receive a surgical treatment for esophageal cancer
In-hospital mortality	3.9	(0.6–7.3)	….with 2.5 % less risk of in-hospital mortality
Persistent GI symptoms	9.5	(2.4–16.6)	….with 30 % less risk of persistent GI symptoms
Morbidity	5.8	(1.2–10.4)	….with 20 % less risk of morbidity
Hospital volume	8.1	(4.0–12.2)	….in a high-volume hospital instead of a low-volume hospital

The confidence intervals were determined using the Delta Method

*GI* gastrointestinal, *CI* 95 % confidence interval

## Discussion

Risk of in-hospital mortality, risk of persistent GI symptoms, chance of 5-year survival, risk of surgical and non-surgical complications, and hospital volume of esophageal cancer surgery all influenced patients’ preferences for surgical management of esophageal cancer. Patients accepted a less effective surgical treatment or a higher risk of persistent GI symptoms, if esophagectomy took place in a higher volume hospital with a lower risk of in-hospital mortality and morbidity. Patients were willing to trade-off 9.5 % (CI 2.4–16.6 %) 5-year survival chance to obtain a surgical treatment with 30 % lower long-term absolute risk of GI symptoms.

Another DCE investigated preferences of patients towards surgery for esophagogastric cancer in the UK and found that long-term treatment outcomes (i.e., quality of life and cure rate) outweighed attributes such as hospital type and a surgeon’s reputation [16]. These results are in line with our findings, which showed that especially persistent GI symptoms and 5-year survival had a relatively large impact on patients’ preferences for surgical management of esophageal cancer. Our finding that patients were prepared to give up life expectancy to avoid side effects of medical intervention was also found by a DCE which focused on patients’ preferences for the management of non-metastatic prostate cancer [17].

Patients included in our study showed preference heterogeneity for several aspects of surgical management of esophageal cancer. Awareness of differences in personal values regarding surgical management is hence important. This study underlines the importance of a patient-tailored approach for discussing prognosis and treatment, which may have a positive effect on the decision process and outcomes in individual patients.

The present study had several limitations. First, although the response rate of 68 % is similar to other DCEs performed [18, 19], this response rate is still not optimal. We cannot exclude selection bias, although the respondents did not differ from the non-respondents in age and sex (data not shown) and matched with the demographics of esophageal cancer patients in the Netherlands [1]. Second, all patients included in our study had experienced surgery for esophageal cancer and therefore knew what they were choosing for in the DCE. This strength is, however, also a limitation. This group of patients may be biased based on their personal experience. Therefore, a prospective study including a pre-operative patient population is recommendable to negate the effect of previous experience on patients’ choices. Third, it is not fully clear whether patients associate better outcome with higher volume hospitals, and that other aspects of higher volume hospitals are appreciated by patients. It is also possible that patients more easily understood the concept of a high-volume hospital than better outcomes as expressed numerically (in %) for in-hospital mortality and morbidity. Fourth, our study is to some extent specific to the Dutch context, that is, a large number of people living in a small country. This means people do not need to travel far to find a high-volume center, and this might have impacted the volume outcome trade-off reported. Finally, the external validity could have been improved if patients were included who were treated in different hospitals with different volumes. This should be kept in mind for future research.

In conclusion, this study showed that patients are willing to trade-off some 5-year survival chance to achieve an improvement in early outcomes. Given the preference heterogeneity among participants, the present study underlines the importance of a patient-tailored approach when discussing prognosis and treatment.
